# In vivo immunomodulation of IL6 signaling in a murine multiple trauma model

**DOI:** 10.1007/s12026-022-09319-3

**Published:** 2022-09-24

**Authors:** Tom Malysch, Jens Michael Reinhold, Christopher A. Becker, Katharina Schmidt-Bleek, Christian Kleber

**Affiliations:** 1 Department of Anaesthesiology and Intensive Care Medicine, Brandenburg Medical School MHB, University Hospital Brandenburg, Brandenburg an der Havel, Germany; 2 Center for Musculoskeletal Surgery, Julius Wolff Institute, Charité-Universitätsmedizin Berlin, Corporate Member of Freie Universität Berlin, Humboldt-Universität Zu Berlin, and Berlin Institute of Health, Berlin, Germany; 3 Department of Orthopedic, Trauma, Hand and Reconstructive Surgery, Sana Hospital Lichtenberg, Berlin, Germany; 4 Department of Orthopaedics and Trauma Surgery, Musculoskeletal University Center Munich (MUM), University Hospital, LMU Munich, Berlin, Germany; 5 Department of Orthopaedics, Trauma and Plastic Surgery, University Hospital Leipzig, Leipzig, Germany

**Keywords:** Polytrauma, IL6, sIL6-R, Trans-signaling, Immunomodulation

## Abstract

A significant number of trauma patients die during the ICU phase of care because of a severe immune response. Interleukin-6 (IL6) plays a central role within that immune response, signaling through a membrane-bound (IL6-R) and a soluble IL6 receptor (sIL6-R). IL6 and the sIL6-R can form an agonistic IL6/sIL6-R-complex, activating numerous cells that are usually not IL6 responsive, a process called trans-signaling. We attempted to demonstrate that modulation of the IL6 signaling (classic signaling and trans-signaling) can attenuate the devastating immune response after trauma in a murine multiple trauma model. Mice were allocated to three study arms: sham, fracture or polytrauma. Half of the animals had the application of an IL6-R antibody following an intervention. After a pre-set time, blood samples were analysed for IL6 and sIL6-R serum levels, organs were analysed for neutrophil infiltration and end organ damage was evaluated. IL6 and sIL6-R showed a rapid peak after fracture, and much more markedly after polytrauma. These parameters were reduced significantly by globally blocking IL6 signaling via IL6-R antibody (Mab) application. Shock organ analysis also illustrated significant neutrophil infiltration following polytrauma, which was also abated via IL6-R Mab application. Furthermore, end organ damage was reduced by IL6-R Mab application. The study results prove the regulatory role of IL6 signaling pathways in polytrauma, with haemorrhagic shock being a major trigger of inflammatory response. Modulation of IL6 signaling shows promise in the prevention of adverse events like organ failure following major trauma and might be a target for in vivo immunomodulation to reduce mortality in severely injured patients, but further evaluation regarding classic IL6 signaling and IL6 trans-signaling is needed.

## Introduction

Polytrauma has a bimodal mortality peak. While the majority of traumatic death occurs in the prehospital phase, a second significant mortality peak develops during the intensive care unit (ICU) phase [1]. This means that a significant number of patients, having survived the initial traumatic event, still die after successful resuscitation. In their clinical course, 50% of polytrauma patients develop multiple organ failure (MOF) [2], which is often a result of an overwhelming systemic immune response to severe trauma [3]. The interaction of haemorrhage and tissue trauma, triggering the inflammatory immune response, is fundamental in both the understanding and successful management of major trauma.

**Fig. 1:**
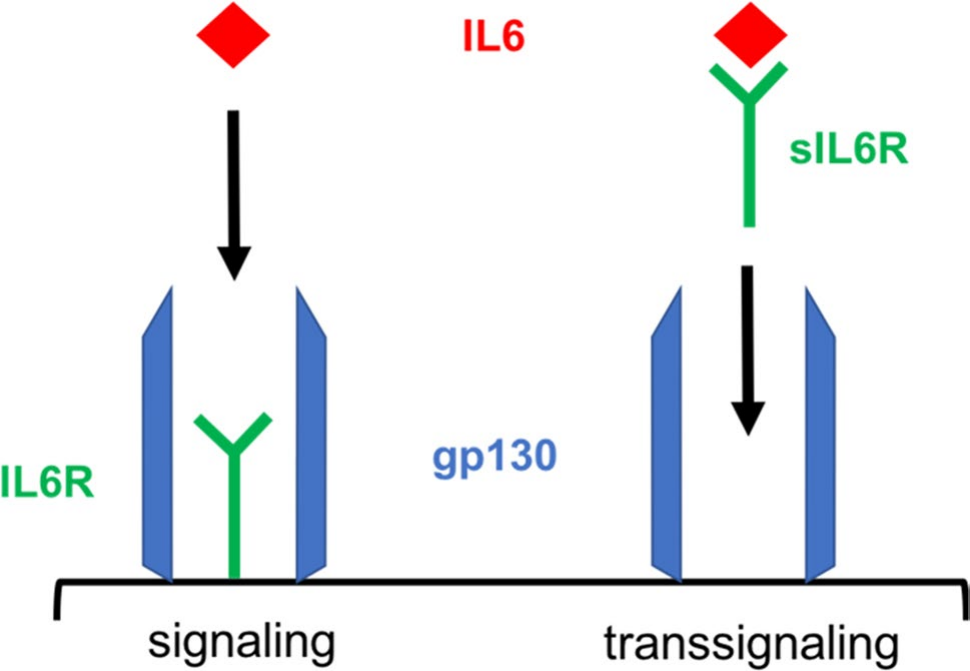
IL6 signaling via membrane-bound IL6 receptor and trans-signaling via soluble IL6 receptor

Severe ischemia due to haemorrhagic shock, as well as direct tissue trauma, induces a significant release of molecules called “danger associated molecular patterns (DAMPs)”, in particular within shock organs [4]. DAMP recognition in those tissues attracts neutrophil granulocytes, which release Interleukin-6 (IL6). IL6 plays a leading role in promoting inflammation via an increased production of acute phase proteins as well as augmentation of the innate and adaptive immune system [5]. IL6 signals through a membrane-bound IL6 receptor consisting of two subunits, namely IL6 receptor alpha (CD126) and the signal transducer protein gp130 (CD130), which is called classic IL6 signaling. Usually, the IL6 receptor is exclusively expressed on liver cells and leukocytes, but due to alternative splicing and shedding, a soluble IL6 receptor (sIL6-R) is formed [6]. Notably, IL6 and the sIL6-R can form an agonistic IL6/sIL6-R-complex, activating cells that express gp130. Since gp130 is a widely spread transmembrane protein, IL6 extends its sphere across the organism, contributing to an overwhelming immune response [7], which can result in “systemic inflammatory response syndrome” (SIRS) [8], contributing to the development of MOF [9]. This mechanism is called IL6 trans-signaling (Fig. 1) and is known to be a factor in sepsis, cancer and autoimmune diseases [10].

Therefore, we hypothesised that IL6 signaling pathways—especially IL6 trans-signaling—have an important role in major trauma as well. Furthermore, blockade of classic IL6 signaling and trans-signaling might help prevent severe immunological aftermath.

## Methods

The research work is based on the dissertation of Dr. Tom Malysch [11].

### Overall study design

Twelve to 14-week-old female C57BL/6 N mice were randomly assigned to the three study groups—“sham” (group I; *n* = 30), “fracture” (group II; *n* = 48) and “polytrauma” (fracture and haemorrhagic shock – group III; *n* = 48)—and received the respective intervention. Twenty-four animals within each study group received a neutralising IL6-receptor antibody (100 µg LEAF™ purified anti-mouse CD126 antibody IL6-Rα chain, BioLegend, San Diego, USA) following the operative procedure by intraperitoneal injection (subgroups Ib, IIb and IIIb), whereas the other animals were left without immunomodulation (subgroups Ia, IIa and IIIa) and did not receive any injection. Following the intervention, the mice received postoperative analgesia (first 3 days) with tramadol-enriched drinking water (25 mg/l). At 6 h, 24 h, 48 h and 21 days, six mice from each subgroup had blood samples taken, the mice were euthanised and their organs were harvested for further histological analysis. The sample size of each subgroup (*n* = 6) was chosen by balancing statistical reasons and considering “reduction” according to the 3Rs concept of animal experimental techniques.

### Murine multiple trauma model

Within our newly proposed long-term survival murine multiple trauma model [12], mice were given general anaesthesia via exposure to volatile isoflurane (1.6 vol%) and administration of subcutaneous buprenorphine (0.03 mg/kg bodyweight). The correct depth of anaesthesia was monitored using respiratory rate and heart rate. Perioperative antimicrobial prophylaxis was achieved by subcutaneous application of clindamycin (8 mg/kg bodyweight). The mice were positioned supine on a thermo-controlled heating plate for maintenance of body temperature and a needle electrocardiogram (ECG) (AD Instruments, USA) was connected.

The induction of haemorrhagic shock was performed via insertion of a PE tube (0.61 mm) through the right common carotid artery. To measure arterial blood pressure (AD Instruments, USA), the animals were bled to a mean arterial blood pressure (MAP) of 35–40 mmHg. MAP was maintained at that level for 60 min. For shock termination, the animals received Ringer’s solution at a volume equal to 1.5 times the shed blood volume within 20 min. After the removal of the catheter, the mice were transferred into a right lateral position for the performance of femoral osteotomy and closed tibia fracture. Afterwards, both fractures (femur and tibia) and the corresponding fixations were radiographically verified in two planes.

According to prior randomisation, blood was taken via intracardial puncture at a pre-set time after the intervention; the mice were euthanised; and the organs were harvested.

### Blood sampling and ELISA

The IL6 serum concentrations were quantified in doublets by ELISA (dilution 1:2; Mouse IL-6 Quantikine Immunoassay M6000B; R&D Systems, Wiesbaden-Nordenstadt, Germany). The sIL6-R serum levels were measured by ELISA in triplets (dilution 1:32; Mouse IL-6 R alpha DuoSet DY1830; R&D Systems). These samples (dilution 1:32) had previously been validated by spike and recovery test (100%, R&D Systems). ELISA readout was performed within 30 min of finishing the assay using a microplate reader at 450 nm with correction at 570 nm and evaluated with Microplate Manager Version 5.2 software (Bio-Rad Laboratories, Inc., Hercules, CA).

The trans-signaling ratio (TSR) was calculated by the quotient of IL6 (pg/ml) over sIL6-R (ng/ml). Therefore, the measured sIL6-R serum levels (pg/ml) were multiplied by a factor of 10^−3^.

### Histology of shock organs

Shock organs were split in half, and one part was prepared for paraffin embedding utilising the 4%-paraformaldehyde solution for 48 h at room temperature (RT) immediately after harvesting, followed by a 45 min tap water flush, and lastly storage using 70% ethanol solution at 4 °C until further processing, which was performed using an automated tissue processor (Leica TP1020, Leica Biosystems, Germany). For slide preparation, a microtome was used, producing 5 µm sections. Subsequently, Mayer’s haematoxylin/eosin (HE) stain was used for paraffin-embedded and sectioned lungs, kidneys, livers and spleens.

The second parts of previously split shock organs were prepared for cryo embedding (frozen section embedding) utilising the 4%-paraformaldehyde solution for 2 h at 4 °C immediately after harvesting, followed by a 10%-, 20%- and 30%-sugar solution for 24 h each, at 4 °C. Afterwards, organs were embedded into SCEM medium (SECTION-LAB Co. Ltd, Japan) and immersed into n-hexane, which itself was immersed into cold acetone, prepared with dry ice. Frozen samples needed to be stored immediately at − 80 °C. For cryo-sectioning, a refrigerated microtome was used at − 19 °C, producing 5 µm sections onto charged adhesion slides. Neutrophil leukocytes in cryo-embedded shock organs were stained using a Ly6G antibody (Ly6G/GR1 Mouse AM26331PU-N, Host: Rabbit, Acris Antibodies).

Initially, paraffin-embedded sections of livers, kidneys, spleens and lungs of the fracture group without immunomodulation (group IIa) and polytrauma group without immunomodulation (group IIIa) were analysed at 24 h with HE stain. For further evaluation, cryo-embedded sections of livers of all study groups were analysed utilising Ly6G stain for neutrophil infiltration. Counts were readjusted to the organ size (cells per cm^2^).

### Macroscopic lung analysis

To estimate extravascular lung water as a surrogate parameter for acute lung injury [13, 14], the right lung of each mouse was withheld from histological processing and was put into a warming cupboard at 37 °C for 3 days. Lung weight was measured before and after the warming phase, to calculate the net difference (delta lung weight), representing the vaporised lung water.

### Statistics

Statistical analysis was performed using IBM SPSS Statistics, Version 24 (IBM, USA). For statistical evaluation, the Mann–Whitney *U* test was used. Data is displayed as the median and interquartile range (IQR) for non-parametric data. Statistical significance was assumed with a *p*-value of 0.05 or less. No mice of the initial sample size were excluded from the evaluation.

## Results

### IL6 and sIL6-R blood levels

**Fig. 2:**
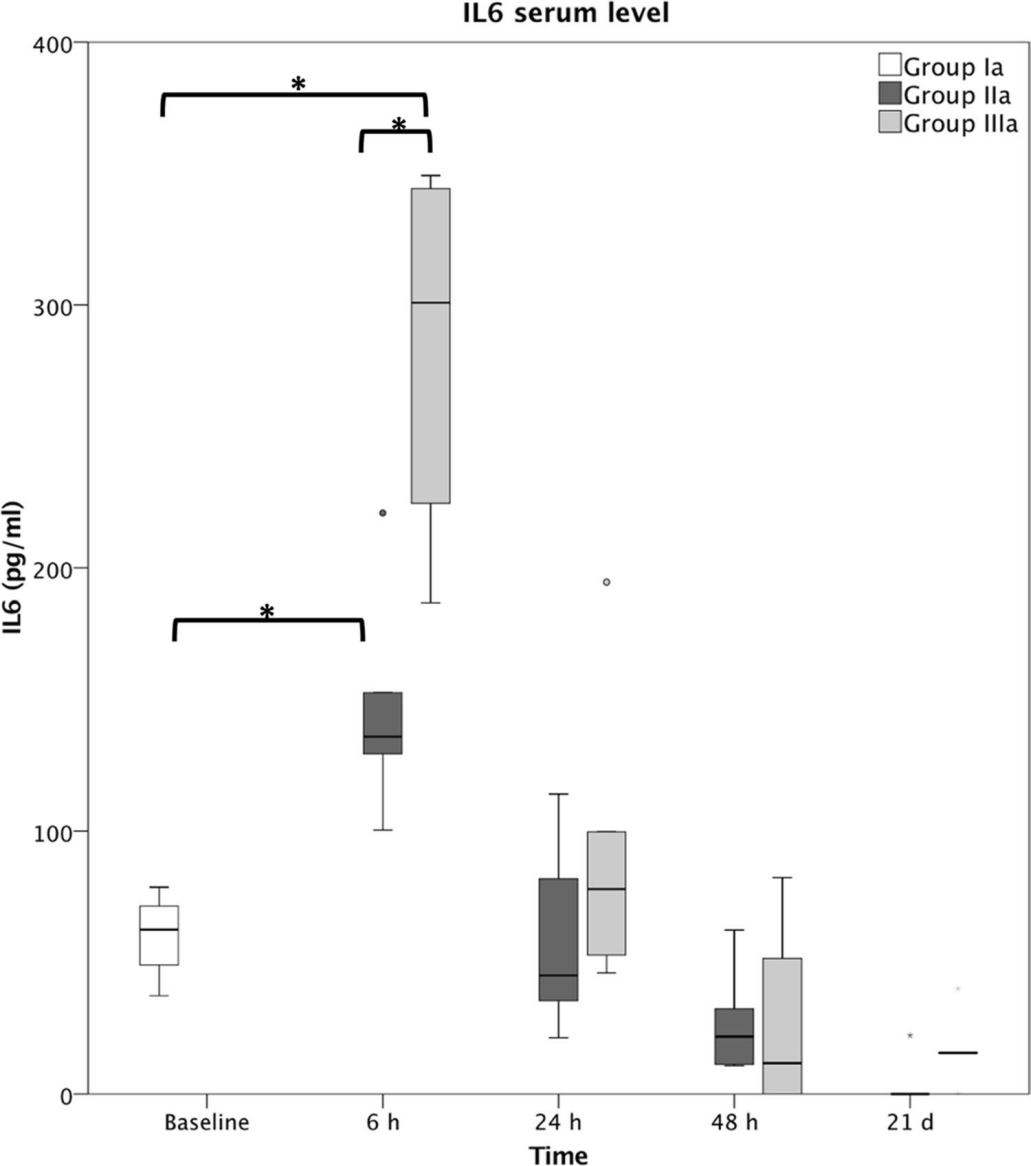
IL6 serum levels following fracture without immunomodulation (group IIa) and polytrauma without immunomodulation (group IIIa). Group Ia represents sham mice. * represents *p* ≤ 0.05

IL6 serum levels (pg/ml), as displayed in Fig. 2, were significantly elevated at 6 h post trauma (sham 62.6 ± 17.1; fracture 135.9 ± 40.6; polytrauma 300.8 ± 75.8) and were returned to baseline from 24 h onwards, without a relevant difference to sham (Ia). Within the polytrauma group (IIIa), the IL6 peak was significantly higher than in the fracture group (IIa).

sIL6-R serum levels (pg/ml) at 6 h and 24 h after fracture (IIa) were significantly elevated from baseline (sham 4573.5 ± 3431.8; IIa at 6 h 9914.5 ± 2572.2; IIa at 24 h 10,360.0 ± 2208.1), declining to baseline at 48 h and 21 days. The same dynamics were applied to the polytrauma group (IIIa at 6 h 10,640.1 ± 3435.7; IIIa at 24 h 9314.5 ± 6314.4).

**Fig. 3:**
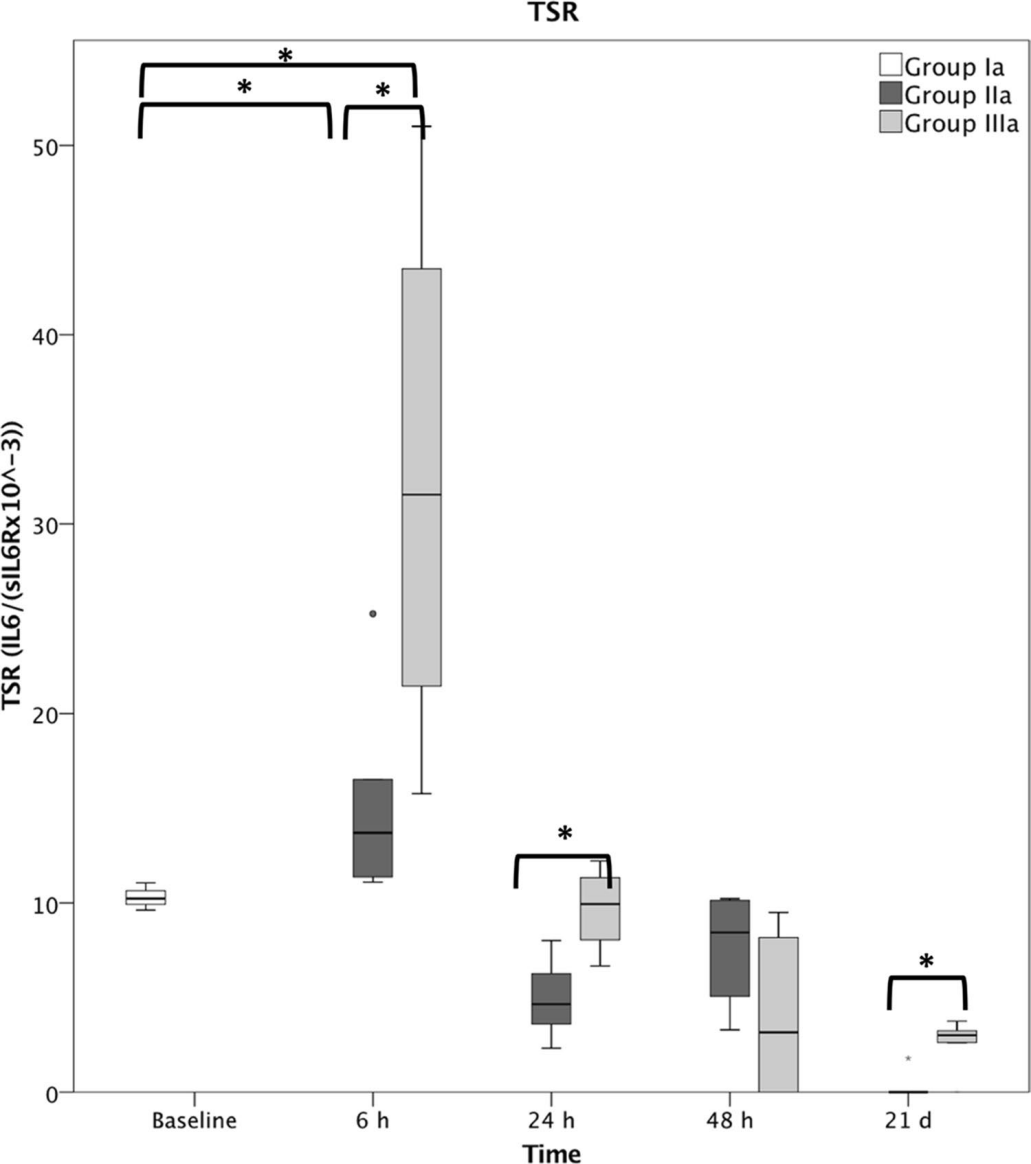
Trans-signaling ratio (TSR) following fracture without immunomodulation (group IIa) and polytrauma without immunomodulation (group IIIa). Group Ia represents sham mice. * represents *p* ≤ 0.05

The TSR (Fig. 3) at 6 h showed a significant change from baseline (sham 10.2 ± 0.7; fracture 13.7 ± 5.3; polytrauma 31.6 ± 14.9), showing a significant difference between the fracture (IIa) and polytrauma (IIIa) group at 6 h, 24 h (fracture 4.6 ± 2.0; polytrauma 9.9 ± 2.3) and 21 days (fracture 0.0 ± 0.5; polytrauma 3.0 ± 1.3). Application of IL6-R Mab in polytrauma (IIIb) led to a decrease in TSR at 6 h and significantly at 24 h (4.1 ± 2.1).

### Shock organs

**Fig. 4:**
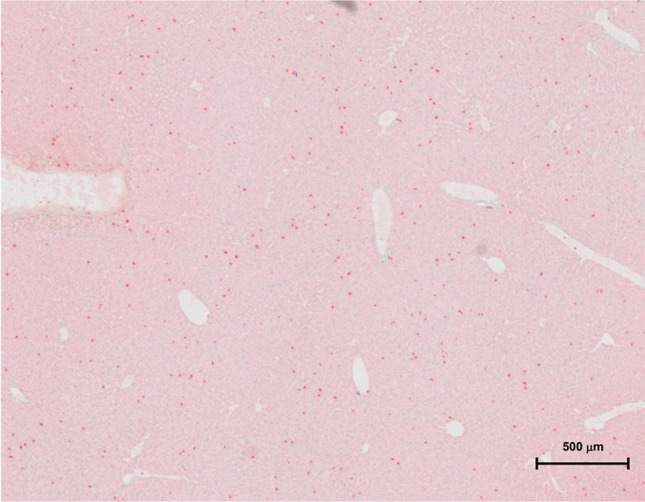
Liver Ly6G stain of group IIIa at 6 h (polytrauma without immunomodulation)

**Fig. 5:**
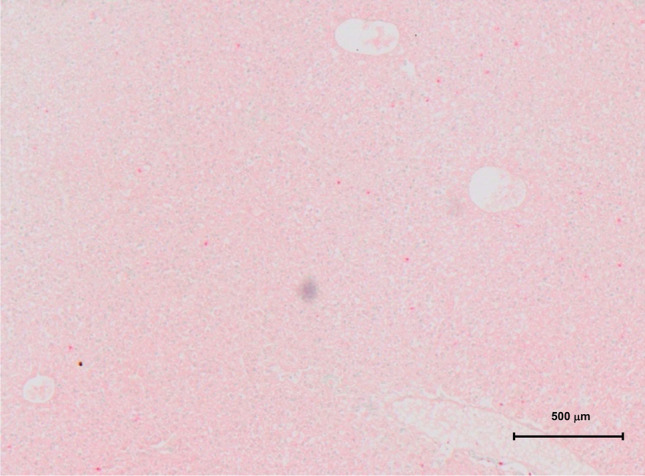
Liver Ly6G stain of group IIIb at 6 h (polytrauma with immunomodulation)

**Fig. 6:**
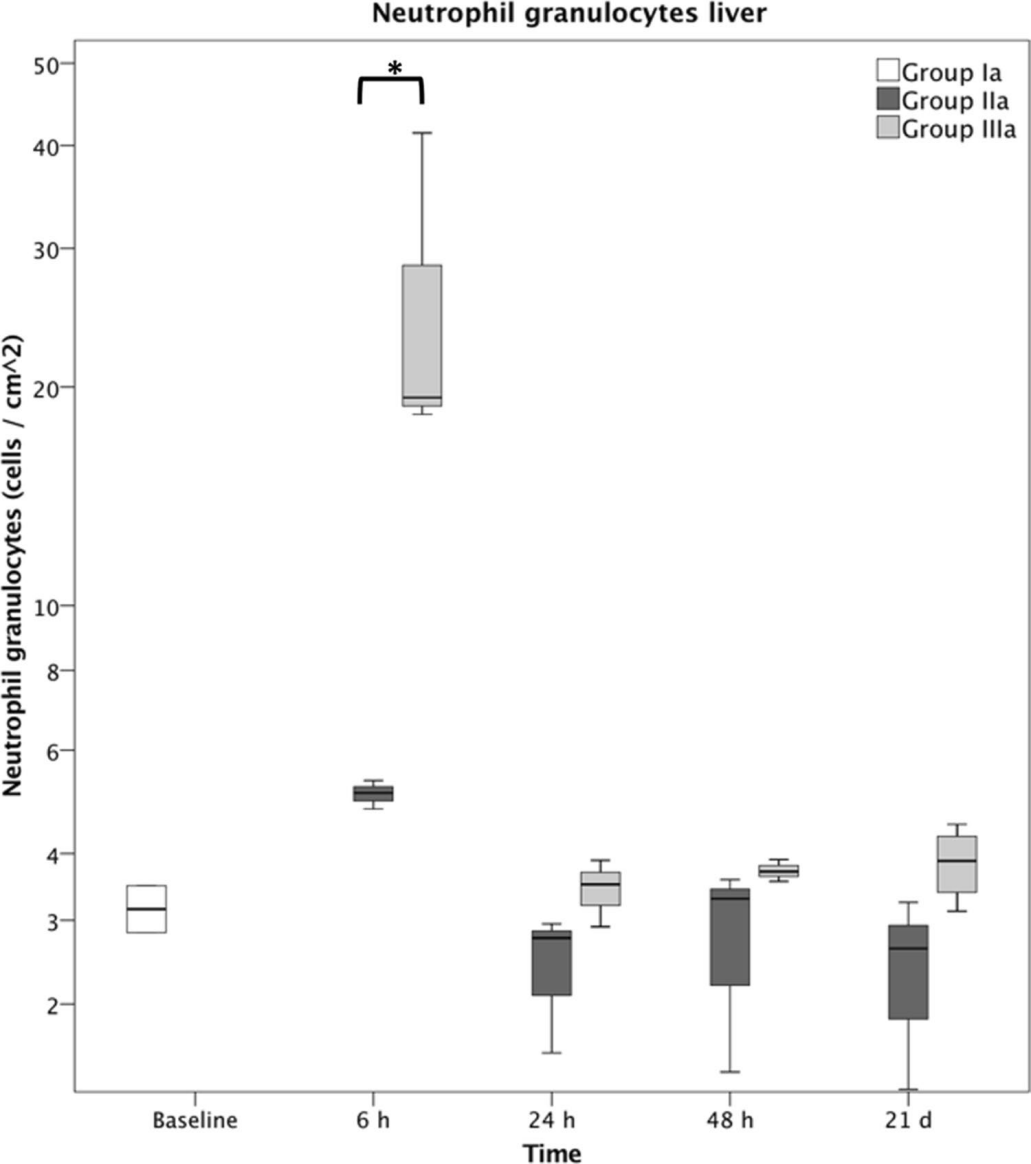
Neutrophil granulocytes in the liver following fracture without immunomodulation (group IIa) and polytrauma without immunomodulation (group IIIa). Group Ia represents sham mice. * represents *p* ≤ 0.05

**Fig. 7:**
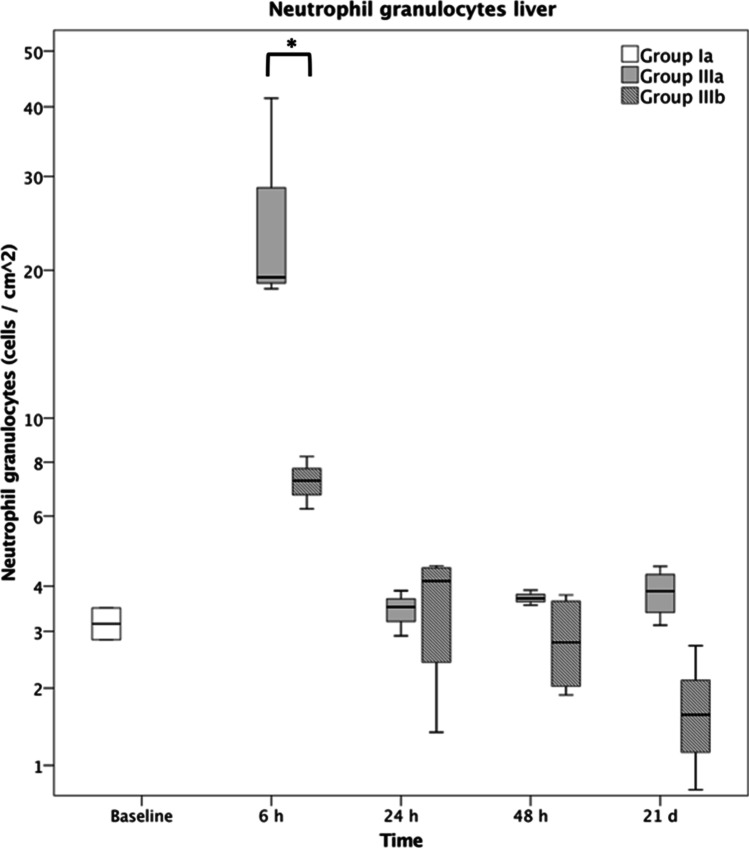
Neutrophil granulocytes in the liver following polytrauma with immunomodulation (group IIIb) and without immunomodulation (group IIIa). Group Ia represents sham mice. * represents *p* ≤ 0.05

Overall, an increased accumulation of neutrophil granulocytes within all shock organs was seen in the polytrauma group (IIIa) compared to the fracture group (IIa). Additional analysis of the liver tissue revealed a significantly higher neutrophil count (cells per cm^2^) at 6 h after polytrauma (IIIa) than after fracture only (IIa) (sham 3.2 ± 0.3; fracture 5.1 ± 0.3; polytrauma 19.4 ± 13.0). From 24 h onwards, the neutrophil count returned to baseline, without any significant difference between the fracture and polytrauma groups. The elevated neutrophil count at 6 h after polytrauma was significantly reduced (7.3 ± 1.0) after application of IL6-R Mab (IIIb), as seen in Figs. 4 and 5. The corresponding data is displayed in Figs. 6 and 7.

**Fig. 8:**
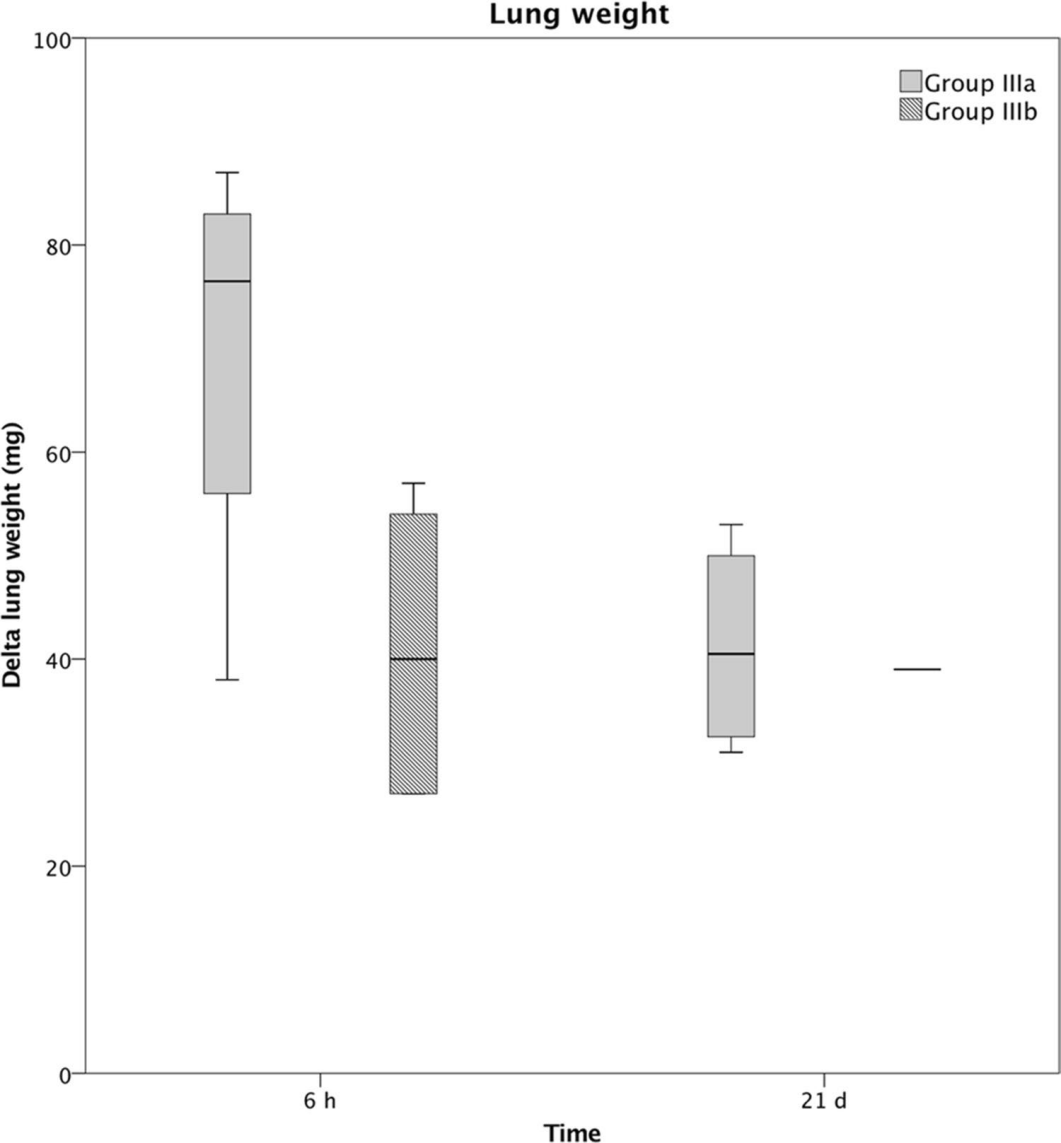
Difference in lung weight following polytrauma with and without immunomodulation (groups IIIa and IIIb). * represents *p* ≤ 0.05

The difference in lung weight (mg) at 6 h and 21 days was significantly higher in the polytrauma group (IIIa at 6 h 77 ± 22; IIIa at 21 days 41 ± 10) than in the fracture group (IIa at 6 h 0 ± 0; IIa at 21 days 0 ± 0). Following the application of IL6-R Mab, the difference in lung weight at 6 h within the polytrauma group (IIIb) decreased significantly (40 ± 14), as shown in Fig. 8.

## Discussion

### Serum parameters

Our murine multiple trauma model reproduced the known effect that trauma causes an increase of IL6 serum levels within 6 h of trauma. Furthermore, the higher the trauma load, the more IL6 increased. These kinetics match data from human analysis [10]. sIL6-R serum levels were increased at 6 h and 24 h after trauma; therefore, trauma probably intensifies the shedding and splicing process that set up the soluble IL6 receptor. Importantly, alternative splicing of IL6-R mRNA has only been described in humans; therefore, in mice, only shedding seems to contribute to that. In our human pilot study, however, we observed a decrease in sIL6-R within the trauma patients compared to healthy individuals [10]. Presumably, human patients had a consumption of sIL6-R within the trans-signaling process, whereas in our murine trauma model, sIL6-R formation might be induced more than it was consumed by trans-signaling, considering the massive haemorrhagic shock that was induced. On the other hand, the soluble form of gp130 (sgp130), known to interact with IL6 and sIL6-R serum levels, was not measured within these data, but might play a relevant role in understanding these serum parameter dynamics [15].

The TSR, as the quotient of IL6 and sIL6-R serum levels, was introduced by our clinical pilot study to obtain a single value representing the trans-signaling process [12]. At 6 h and 24 h after trauma, TSR showed significant group differences between polytrauma, fracture and sham mice, matching our findings from human data, when TSR was able to distinguish between survivors and non-survivors at 6 h and 24 h after trauma [10]. Application of IL6-R Mab, blocking classic IL6 signaling and trans-signaling, reduced the TSR at these important “mortality checkpoints” 6 h and 24 h after trauma.

Even though trauma in general led to a rise in the above-mentioned serum levels and thus TSR, one needs to take into account that the musculoskeletal trauma was the same between the fracture group and the polytrauma group. Therefore, it would appear to be the haemorrhagic shock primarily, in combination with the prolonged operative procedure, that is causative of the significant increase in TSR in comparison to the “fracture only” animals.

### Shock organs

Comparing neutrophil granulocytes within shock organs after fracture and polytrauma revealed that adding haemorrhagic shock to a musculoskeletal trauma led to increased infiltration of those cells, consistent with the ELISA data indicating increased inflammation. The peak of neutrophil infiltration matches the serum parameter peak at 6 h after trauma and already returns towards the baseline at 24 h. Therefore, we postulate that if a patient is kept from another immunological hit after trauma, such as extended surgical measures, the effects on the shock organs, represented by the degree of neutrophil infiltration, seem to decrease as quickly as the systemic immune response, illustrated by the reduction of IL6 serum levels from 6 to 24 h after trauma.

The IL6-R Mab application led to a significant prevention of neutrophil infiltration within the expected peak at 6 h, as well as decreasing IL6 serum levels and TSR at the same time point. Notably, TSR levels within our human data, especially at 6 h, were associated with the degree of organ failure and mortality, most likely associated with the degree of neutrophil infiltration, as shown within the murine data. Thus, reduction of the neutrophil granulocyte peak (and TSR) may potentially lead to less organ failure, as has previously been described for acute lung injury and liver injury [16, 17]. However, in sepsis, global blockage of classic IL6 signaling and trans-signaling via IL6-R Mab application was not beneficial, while only selective inhibition of IL6 trans-signaling was [18]. Interestingly, classic IL6 signaling seems to be of profound importance for the organisms’ control of infection, whereas restricting IL6 trans-signaling did not restrain this ability [19]. Additionally, fracture healing after trauma was shown to be improved by selective inhibition of IL6 trans-signaling, but not by global inhibition of IL6 signaling [20]. Nevertheless, our data suggests that in polytrauma (mice), global blockage of IL6 signaling appears to be useful regarding organ failure.

In keeping with our results, we saw an increased amount of lung water within our polytrauma mice, demonstrating measurable end organ damage. Therefore, our results from serum level parameter data and neutrophil infiltration counts seem to translate into objectifiable organ failure. Using immunomodulation via IL6-R Mab application prevented increased lung water within the polytrauma mice.

## Conclusion

In polytrauma, an understanding of the interaction of haemorrhage and tissue trauma in triggering an immune response would appear fundamental in improving mortality rates. Our data revealed that IL6 as well as sIL6-R showed a rapid peak within 6 h after trauma and are thus potentially of value before other serum biomarkers in the clinical context [21].

Murine shock organ analysis coherently illustrated a significant neutrophil infiltration within the 6 h after polytrauma. The neutrophil peak returned to baseline at 24 h, potentially allowing recovery from organ injury when another inflammatory stimulus (second hit) is prevented, supporting the idea of damage control resuscitation/surgery (DCR/DCS).

The initial neutrophil peak, considered responsible for organ failure and immune paralysis [22, 23], was prevented by globally blocking classic IL6 signaling and trans-signaling via IL6-R Mab application, as demonstrated in the liver. That reduction of inflammation led to a notable reduction of end organ damage, as demonstrated within the lungs.

Our results, therefore, underscore the important role of IL6 signaling pathways in polytrauma, with haemorrhagic shock being a major trigger of the inflammatory response. Importantly, the roles of classic IL6 signaling and IL6 trans-signaling after trauma must be recognised individually regarding the context (bone healing vs. organ failure) and severity of trauma (minor vs. major), and this, therefore, needs further evaluation.
